# Exosomal miR-22-3p Derived from Chronic Rhinosinusitis with Nasal Polyps Regulates Vascular Permeability by Targeting VE-Cadherin

**DOI:** 10.1155/2020/1237678

**Published:** 2020-11-12

**Authors:** Wei Zhang, Ting Zhang, Yongbing Yan, Jie Zhang, Yong Zhou, Yinyin Pei, Li Yao, Bo You, Jing Chen

**Affiliations:** ^1^Institute of Otolaryngology Head and Neck Surgery, Affiliated Hospital of Nantong University, Nantong, Jiangsu, China; ^2^Department of Otolaryngology Head and Neck Surgery, Affiliated Hospital of Nantong University, Nantong, Jiangsu, China; ^3^Department of Otolaryngology Head and Neck Surgery, The Third People's Hospital of Nantong, Jiangsu, China

## Abstract

**Background:**

The abnormal vascular permeability is associated with the formation of chronic rhinosinusitis with nasal polyps (CRSwNP). Previously, our study demonstrated that the nasal lavage fluid- (NLF-) derived exosomes from CRSwNP can promote the vascular permeability of human umbilical vein endothelial cells (HUVECs). miR-22-3p, a specific differentiated miRNA, is reported to regulate microvessels in some diseases. This study is purposed to explore the impact of exosomal miR-22-3p derived from CRSwNP on vascular permeability and identify the underlying targets.

**Methods:**

Exosomes were extracted from NLF of 26 CRSwNP patients and 10 control patients. Quantitative real-time PCR (qRT- PCR) was applied to evaluate the relative level of exosomal miR-22-3p. The impact of exosomal miR-22-3p on HUVECs was assessed by permeability assays in vitro. The potential molecular targets of miR-22-3p were investigated by applying such technologies as dual-luciferase reporter assay and western blot.

**Results:**

miR-22-3p was upregulated in NLF-derived exosomes from CRSwNP. Exosomal miR-22-3p derived from CRSwNP enhanced the tubule permeability of HUVECs. Vascular endothelial- (VE-) cadherin (*CDH5*) was identified as a direct target of miR-22-3p. miR-22-3p regulated the vascular permeability by targeting VE-cadherin in HUVECs.

**Conclusions:**

Exosomal miR-22-3p derived from NLF of CRSwNP plays an important role in regulating vascular permeability by targeting VE-cadherin.

## 1. Introduction

Chronic rhinosinusitis (CRS) is characterized by the chronic inflammation of the paranasal sinus mucosa that persists for at least 12 weeks [1]. At present, CRS affects 8% of the population in China, which not only reduces the quality of life but also causes a considerable socioeconomic burden [2]. CRS is classified into 2 types depending on the absence or presence of nasal polyps (NPs): chronic rhinosinusitis without nasal polyps (CRSsNP) and chronic rhinosinusitis with nasal polyps (CRSwNP) [3]. One of the significant pathological characteristics exhibited by CRSwNP is interstitial edema with inflammatory cell infiltration. In some studies, it has been revealed that the increase in capillary and basilar membrane permeability can lead to edema of the mucosa and polyp growth [4, 5]. However, the underlying mechanism of polyp formation in CRSwNP patients remains unclear.

Exosomes are 30-150nm membrane vesicles involved in intercellular communication [6]. Exosomes exist in a variety of body fluids, such as serum, urine, and breast milk [7–10]. In our previous studies, it has been discovered that exosomes are also existent in nasal lavage fluid (NLF), and that NLF-derived exosomes from CRSsNP can increase the vascular permeability of human umbilical vein endothelial cells (HUVECs) [11]. Exosomes are capable to affect the function of the recipient cell by transferring proteins and genetic materials such as mRNAs and microRNAs (miRNAs) between cells [12, 13]. Thus, the role of miRNAs in exosomes should be taken into account.

MicroRNAs (miRNAs) are small, 18-25 nucleotide, noncoding small RNAs that mediate the inhibition of translation and lower protein levels by binding to the 3′-untranslated region of target mRNAs. As the basis of gene regulatory networks, miRNAs are involved in a variety of different biological and pathological processes, for example, growth and development, stress response, cell proliferation, differentiation, apoptosis, and tumorigenesis [14]. The presence of miRNAs has already been evidenced by the exosomes extracted from certain clinical specimens, such as nasal mucus, NLF, and bronchoalveolar lavage fluids, which confirm that the environmental factors and mediators associated with the inflammatory responses can contribute to the development of such respiratory allergy diseases as allergic rhinitis and asthma [15, 16]. The previous research has also suggested that miRNAs were involved in the development of CRSwNP [17, 18]. However, the changes to exosomal miRNAs in CRSwNP were not mentioned.

As a specific differentiated miRNA with a length of 22 nucleotides, miR-22 is highly conserved in the evolutionary process and is differentially expressed in various diseases [19]. Some studies have demonstrated that miR-22 can affect the progression of some diseases by regulating microvessels [20, 21]. Nevertheless, the impact of miR-22 on the microvascular of CRSwNP has been barely reported. In this study, an investigation was conducted into the impact of exosomal miR-22-3p derived from CRSwNP on vascular permeability, and an attempt was made to identify the underlying targets.

## 2. Materials and Methods

### 2.1. Collection of Clinical Specimens

Twenty-six patients with CRSwNP were enrolled in this study at the Affiliated Hospital of Nantong University (Nantong, China). The diagnosis of CRSwNP was according to the European Position Paper on rhinosinusitis and nasal polyp guidelines [1]. Each patient underwent routine examination before the operation, including a medical inquiry, physical examination, nasal endoscopy, a computerized tomography (CT) scan, and skin prick test (SPT). No patient had a history of ciliary dysfunction, cystic fibrosis, autoimmune disease, or immunodeficiency, and none had received topical and/or systemic nasal steroid treatment at least 3 weeks before surgery. Ten patients with deviated nasal septum (DNS) were recruited as the control group. Nasal polyp and inferior turbinate (IT) tissues were collected during surgery and were stored at -80°C until used. This study was approved by the Medical Ethics Committee of the Affiliated Hospital of Nantong University (Nantong, China), and written informed consent was obtained from all patients. Subject characteristics are shown in Table 1.

### 2.2. Cell Culture

The primary human nasal epithelial cells, pHNECs, were isolated by enzymatic method [22]. The samples of human healthy mucosa from nasal deviation patients were obtained during endoscopic sinus surgery. Immediately obtaining the specimens, they were rinsed several times and digested with Proteinase K (YEASEN, Shanghai, China) in serum-free medium DMEM incubated at 37°C, and then the tissue homogenate was filtered and centrifuged to collect the cell pellets. Finally, pHNECs were resuspended in serum-free Airway Epithelial Cell Growth-Medium (BEGM, PromoCell, Germany) and cultured in a petri dish under the condition with 95% humidified air and 5% CO_2_ at 37°C. After, pHNECs reached about 80% confluency and were changed to air-liquid interface (ALI) culture.

HUVECs (ScienCell Research Laboratories, Inc., San Diego, CA, USA) were cultured in DMEM low glucose (HyClone, Logan, UT, USA) and incubated at 37°C containing 5% CO_2_.

### 2.3. Isolation and Purification of Exosomes

NLF was collected from NP and DNS patients, respectively, and detailed methods can be found in our previous work [11]. Exosomes were isolated by differential ultracentrifugation as we have described before [11]. Briefly, the supernatants from both NLF and pHNEC culture medium were centrifuged at 6,000 × g for 30 min and then 10,000 × g for 60min at 4°C, followed by ultrafiltration (0.2*μ*m filter; Sarstedt, Nümbrecht-Rommelsdorf, Germany) and qEV size-exclusion columns (Izon Science, Christchurch, New Zealand). Thereafter, the supernatant was then ultracentrifuged at 100,000 × g for 60min at 4°C (Type 90 Ti Rotor; Beckman Coulter, Inc., Brea, CA, USA) to pellet the exosomes. The exosome pellets were then washed using PBS for cell experiments.

### 2.4. Identification of Exosomes

The exosomes were examined in a Transmission Electron Microscope (JEM-1230; JEOL, Ltd., Tokyo, Japan). Western blot was used to detect the expression of exosomal specific markers. Nanoparticle tracking analysis (NTA) provides high resolution of particle concentration, size, and aggregation measurements. Appropriate exosome concentrations were used to assess the size distribution by Zeta View (Particle Metrix GmbH, Meerbusch, Germany).

### 2.5. Immunohistochemistry (IHC)

To validate the expression of VE-cadherin in tissues, after being baked in a 60°C incubator for at least 4 hours, Paraffin-embedded NP and control tissue sections were deparaffinized, rehydrated, and incubated with citrate buffer for antigen retrieval and heated in an autoclave. Then, sections were incubated with the primary antibody overnight at 4°C, followed by incubation with a HRP-labeled secondary antibody for 30min. All samples were visualized by DAB Detection Kit (ZSGB, Beijing, China) and counterstained the sections with 10% hematoxylin. The expression level was visualized by ZEISS optical microscope (Germany), and the final evaluation of staining was scored by the staining intensity and distribution. The antibodies we used are as follows: anti-VE-cadherin antibody (1 : 200, Abcam). All the experiments were repeated three times.

### 2.6. Western Blot Analysis

Antibodies against flotillin-1 (1 : 1000, Abcam), CD63 (1 : 500, Proteintech), GAPDH (1 : 500, Santa), VE-cadherin (1 : 1000, Abcam), CD9 (1 : 2000, CST), ALIX (1 : 1000, Abcam), TSG101 (1 : 1000, Abcam), and GM130 (1 : 1000, Abcam) were used in western blot as previously described [11].

### 2.7. RNA Isolation and Quantitative Real-Time PCR (qRT- PCR) Analysis

Briefly, cellular RNA was isolated with TRIzol reagent (Invitrogen), while exosomal miRNA was extracted using the Total Exosome RNA Kit (Ambion) and MirVana RNA isolation kit (Ambion) according to recommendations. GAPDH and U6 served as an endogenous control to normalize the expression level of miR-22-3p and VE-cadherin, respectively. The 2^−*ΔΔ*Ct^ method [23] was utilized to evaluate relative expression levels. The primer sequences were designed as follows: miR-22-3p Forward: 5′-AAGCUGCCGUUGAAGAACUGU-3′; Reverse: 5′-GTGCAGGGTCCGAGGT-3′; U6 Forward: 5′-CTCGCTTCGGCAGCACA-3′; Reverse: 5′-AACGCTTCACGAATTTGCGT-3′; VE-cadherin Forward: 5′-CCGCTCGAGACCAATTCCTATAACCTTC-3′; Reverse: 5′-TATGCGGCCGCTTCCCCATGAGGCTCTCTG-3′; GAPDH Forward: 5′-CAGGAGGCATTGCTGATGAT-3′; Reverse: 5′-GAAGGCTGGGGCTCATTT-3′.

### 2.8. Plasmid Transfection

Synthetic miR-22-3p mimic/NC/inhibitor, small hairpin RNA (shRNA) for VE-cadherin (pcDNA3.1 vector inserted the full-length CDS sequence of VE-cadherin), and their negative controls were supplied by GeneChem (Shanghai, China). About 50nM/L plasmid was transfected via Lipofectamine 2000 (Invitrogen, Carlsbad, CA) into pHNECs or HUVECs according to the manufacturer's directions. After 48 hours of transfection, we firstly observed GFP-fluorescence by using an inverted microscope (Zeiss, Gottingen, Germany), and then qRT-PCR was used to further verify the transfection efficiency. The cells were harvested and utilized to subsequent experiments.

### 2.9. Uptake Experiment

To observe the cellular uptake of exosomes, purified exosomes labeled using PKH-67 labeling kit (Sigma-Aldrich, St. Louis, MO, USA) were cocultured with HUVECs. After 1 h, HUVECs were fixed and stained with Hoechst. Photographs were taken with a TCS SP-5 confocal microscope (Leica Microsystems, Wetzlar, Germany).

### 2.10. In Vitro Permeability Assay

As we have mentioned before [11], briefly, HUVECs were seeded on a transwell chambers (0.4*μ*m pore size; Costar, Corning, NY, USA) in 24-well plates and grown until reaching confluence. Then, fluorescein isothiocyanate- (FITC-) dextran (1mg/ml) (M, 40,000; Thermo Fisher Scientific, Inc., Waltham, MA, USA) was added to the upper chamber. 50*μ*l samples were taken from the lower chamber and measured using a fluorescence plate reader (Lambda Fluoro 320; MWG Biotech, Ebersberg, Germany).

### 2.11. Target Prediction

Candidate targets of miR-22-3p were predicted using TargetScan (http://www.targetscan.org).

### 2.12. Dual-Luciferase Reporter Assay

To examine whether miR-22-3p targets VE-cadherin directly, we constructed wild-type *CDH5* 3′UTR reporter plasmid (*CDH5* WT) and mutated-type *CDH5* 3′UTR reporter plasmid (*CDH5* MUT) with pGL3-promoter vector (Ambion). 293T cells were placed on 24-well plates and cultured in DMEM with 10% fetal bovine serum (FBS) at 37°C in an incubator containing 5% CO_2_ until it has grown to 60% confluence. And then, 293T cells were cotransfected with luciferase plasmids and miR-22-3p mimic or negative control (NC). After 48h transfection, luciferase activities were measured using a Dual-Luciferase Reporter Assay System (Promega) according to the manufacturer's instructions.

### 2.13. Statistical Analysis

The data were presented as mean ± standarderror, and statistical analysis was performed using GraphPad Prism® software. Shapiro-Wilk normality test was used to analyze if the values come from Gaussian distribution. Student's *t*-test and ANOVA were used to analyze statistical significance. The Spearman test was used to determine correlations. ^∗^*p* < 0.05 was considered a statistically significant difference.

## 3. Results

### 3.1. Characterization of NLF-Derived Exosomes from CRSwNP

**Figure 1:**
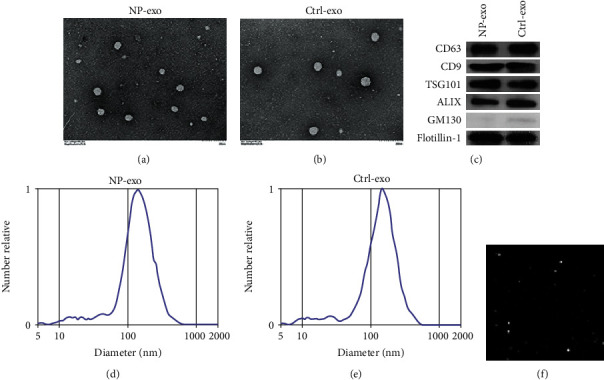
Characterization of NP exosomes. (a, b) Representative electron microscopy image of NP-exo and Ctrl-exo. (c) Western blot analysis of exosomal markers, GM130 was used as a nonexosomal marker; flotillin-1 was used as a loading control. (d–f) Nanoparticle tracking analysis displayed the size distribution of exosomes isolated from NLF.

Firstly, we purified exosomes from the NLF of CRSwNP (NP-exo) and DNS patients (Control-exo) by differential centrifugation. Exosomal lipid bilayer membranes were observed under transmission electron microscopy (TEM), which confirmed the presence of exosomes in the morphology (Figures 1(a) and 1(b)). To confirm the identity of isolated exosomes, exosomal markers were examined. Western blot analysis revealed the expression of exosomal markers such as CD63, CD9, ALIX, and TSG101 [24] in NLF-derived exosomes but absent of GM130 (Figure 1(c)). NanoSight showed the particle size value between 30-150nm (Figures 1(d) and 1(f)). All the results indicated that we successfully isolated exosomes from NLF.

### 3.2. Exosomal miR-22-3p Derived from CRSwNP Enhanced Tubule Permeability of HUVECs

**Figure 2:**
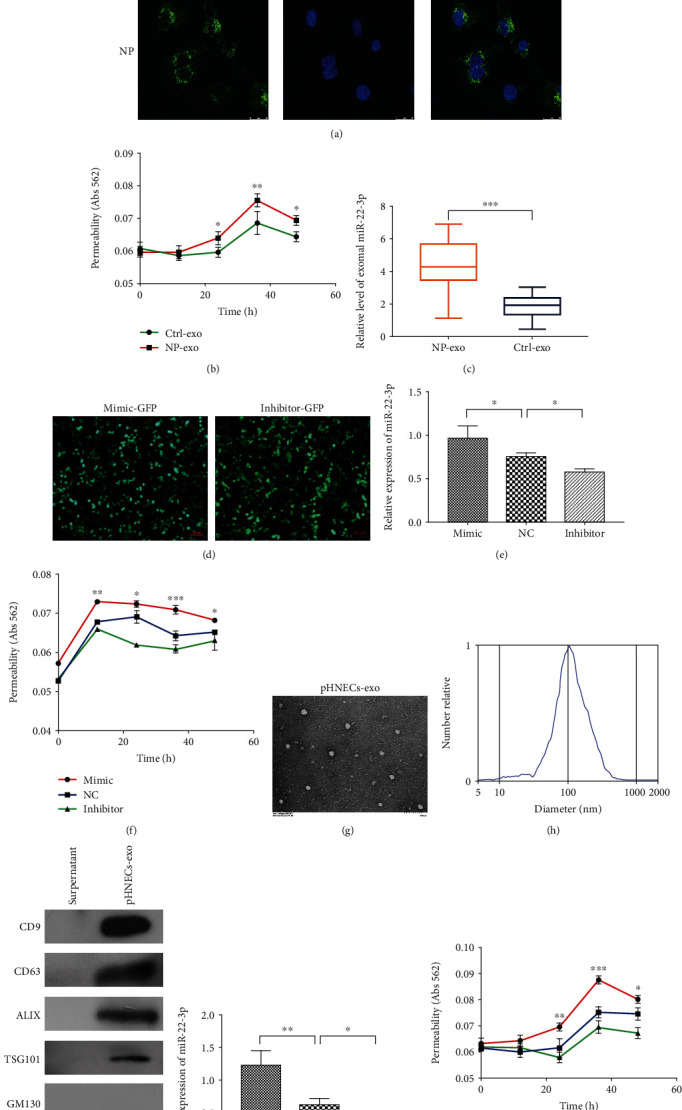
Exosomal miR-22-3p derived from CRSwNP regulates the vascular permeability in vitro. (a) Confocal microscopy analysis of PKH67-labeled NLF-derived exosomes uptaken by HUVECs following coculture for 3h (scale bars, 10*μ*m). Blue: Hoechst staining; green: PKH67-labeled exosomes. (b) Tubule permeability of HUVECs treated with different exosomes was measured by in vitro permeability assay. Ctrl vs. NP. Student's *t* test. ^∗^*p* < 0.05 and ^∗∗^*p* < 0.01. (c) qRT-PCR of miR-22-3p expression in NP-exo and Ctrl-exo. (d) The representative fluorescence figures of HUVECs transfected with miR-22-3p. (e) Transfection efficiency of miR-22-3p was measured by qRT-PCR. One way ANOVA. ^∗^*p* < 0.05. (f) Effects of different levels of miR-22-3p on HUVECs permeability. Mimic vs. NC. Two-way ANOVA. ^∗^*p* < 0.05, ^∗∗^*p* < 0.01, and ^∗∗∗^*p* < 0.001. (g) Representative electron microscopy image of exosomes isolated from supernatant of pHNECs. (h) Nanoparticle tracking analysis of pHNECs-exo. (i) Western blot analysis of exosomal markers. (j) Forty-eight hours after treatment with exosomes isolated from supernatant of transfected pHNECs, miR-22-3p levels of HUVECs were measured by qRT-PCR. One-way ANOVA. ^∗^*p* < 0.05 and ^∗∗^*p* < 0.01. (k) Effects of different levels of exosomal miR-22-3p on HUVEC permeability. Mimic vs. NC. Two-way ANOVA. ^∗^*p* < 0.05, ^∗∗^*p* < 0.01, and ^∗∗∗^*p* < 0.001. Data are presented as mean ± standarddeviation of at least three independent experiments.

Since the development of CRSwNP was associated with abnormal vascular permeability, the effects of exosomes from CRSwNP on the vascular permeability of HUVECs were further evaluated. As shown in Figure 2(a), the incubation of fluorescent NLF-derived exosomes (green) resulted in the transfer of fluorescence to the recipient cells (HUVECs), showing that exosomes could be taken up by recipient cells. In addition, NLF-derived exosomes from CRSwNP were significantly more potent in enhancing the vascular permeability of HUVECs (Figure 2(b)). And then, qRT-PCR was performed to show that miR-22-3p was highly expressed in exosomes from CRSwNP (*n* = 26) than the control group (*n* = 10) (Figure 2(c)). To verify the effect of exosomes on vascular permeability is related to miR-22-3p, we attempted to establish a model system to alter exosomal miR-22-3p expression. Firstly, miR-22-3p mimic, NC, and inhibitor were transfected to HUVECs, respectively (Figures 2(d) and 2(e)). After a series of cellular analyses, we found that the upregulation of miR-22-3p enhanced the vascular permeability of HUVECs, while silencing miR-22-3p expression inhibited the function (Figure 2(f)). Furthermore, pHNECs with high or low miR-22-3p expression were obtained after transfection with miR-22-3p mimic, NC, and inhibitor. Then, exosomes were extracted from the cell supernatant (Figures 2(g)–2(i)); qRT-PCR confirmed that the exosomal miR-22-3p expression significantly changed with respect to transfection; a relatively high exosomal miR-22-3p expression was observed for mimic-treated pHNECs vs. inhibitor-treated cells (Figure 2(j)). After that, HUVECs were cocultured with these exosomes containing different levels of miR-22-3p; subsequent in vitro permeability assay showed that HUVECs cocultured with miR-22-3p-overexpressing exosomes increased the vascular permeability (Figure 2(k)). These results collectively suggest that exosomes contain different levels of miR-22-3p accompanied by potential changes in vascular permeability, and exosomes mediate the transfer of miR-22-3p.

### 3.3. miR-22-3p Directly Targets VE-Cadherin

**Figure 3:**
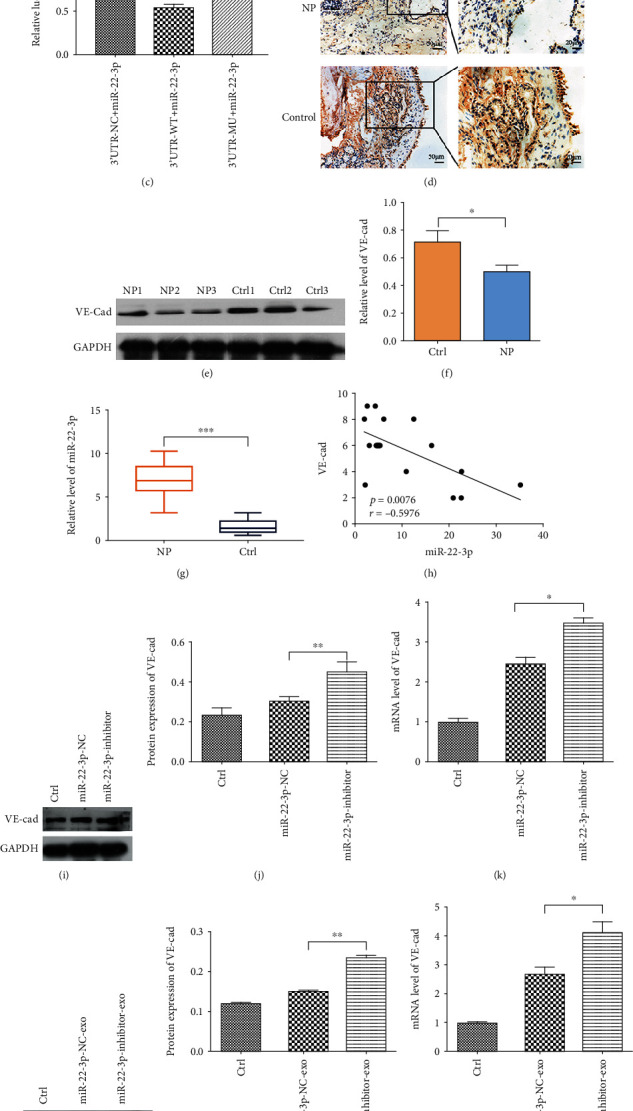
miR-22-3p directly targets VE-cadherin. (a) TargetScan software predicated that *CDH5* (VE-cadherin) was a potential target of miR-22-3p. (b) Luciferase reporter vectors containing WT and MUT *CDH5* 3′UTR were constructed. (c) Luciferase activity was significantly decreased in 293T cells cotransfected with the WT *CDH5* 3′UTR vector and miR-22-3p, but was not significantly affected in cells cotransfected with the MUT *CDH5* 3′UTR vector and miR-22-3p, relative to the control group. One-way ANOVA. ^∗^*p* < 0.05 and ^∗∗^*p* < 0.01. (d) Representative IHC images of VE-cadherin in CRSwNP and IT tissues. VE-cadherin staining was mainly localized in the cytoplasm of cells. (e, f) Western blot of VE-cadherin expression in CRSwNP tissues. Student's *t* test. ^∗^*p* < 0.05. (g) qRT-PCR of miR-22-3p expression in tissues. Student's *t* test. ^∗∗∗^*p* < 0.001. (h) Pearson correlation between miR-22-3p and VE-cadherin expression. Linear correlation. (i, j) Western blot analysis and (k) qRT-PCR were used to measure the expression of VE-cadherin in HUVECs transfected with miR-22-3p-NC or miR-22-3p inhibitor. One-way ANOVA. ^∗^*p* < 0.05 and ^∗∗^*p* < 0.01. (l–n) The expression of VE-cadherin at the protein and mRNA levels in HUVECs, respectively, cocultured with exosomes derived from pHNECs transfected with miR-22-3p-NC or miR-22-3p inhibitor. One-way ANOVA. ^∗^*p* < 0.05 and ^∗∗^*p* < 0.01. Data are presented as mean ± standarddeviation of at least three independent experiments.

As we know, miRNA exerts its biological functions via posttranscriptional gene regulation, so we should first identify the direct targets of miR-22-3p. TargetScan software showed that there were hundreds of potential targets. Several candidate target genes were then selected for possible participation in regulating vascular permeability, including cadherin 5 (*CDH5*, VE-cadherin), cingulin-like 1 (*CGNL1*), cell adhesion molecule 1 (*CADM1*), and cell adhesion molecule 3 (*CADM3*). Among these candidate genes, *CDH5* stood out for the presence of potentially high binding sites (Figures 3(a) and 3(b)). And then, luciferase assays were performed to detect the biologically effective interaction of miR-22-3p and *CDH5* 3′-UTR in 293T cells, showing that luciferase activity in 293T cells cotransfected with WT *CDH5* 3′UTR vector and miR-22-3p mimic was significantly decreased compared to the *CDH5* 3′UTR-NC group, and when the miR-22-3p binding site was mutated in the *CDH5* 3′UTR, luciferase activity was not significantly inhibited by miR-22-3p (Figure 3(c)). Moreover, VE-cadherin was a lower expression in CRSwNP tissue samples vs. control group (IT samples) (Figures 3(d)–3(f)), but miR-22-3p was an overexpression in CRSwNP tissue samples (Figure 3(g)); there was a negative correlation between them (Figure 3(h)). Consistent with the results of luciferase assays, both western blot and qRT-PCR showed that treatment with miR-22-3p-inhibitor (Figures 3(i)–3(k)) or miR-22-3p-inhibitor-exo (Figures 3(l)–3(n)), the expression of VE-cadherin in HUVECs increased. Taken together, these results verified that VE-cadherin is a direct target of miR-22-3p.

### 3.4. miR-22-3p Regulates the Vascular Permeability by Targeting VE-Cadherin in HUVECs

**Figure 4:**
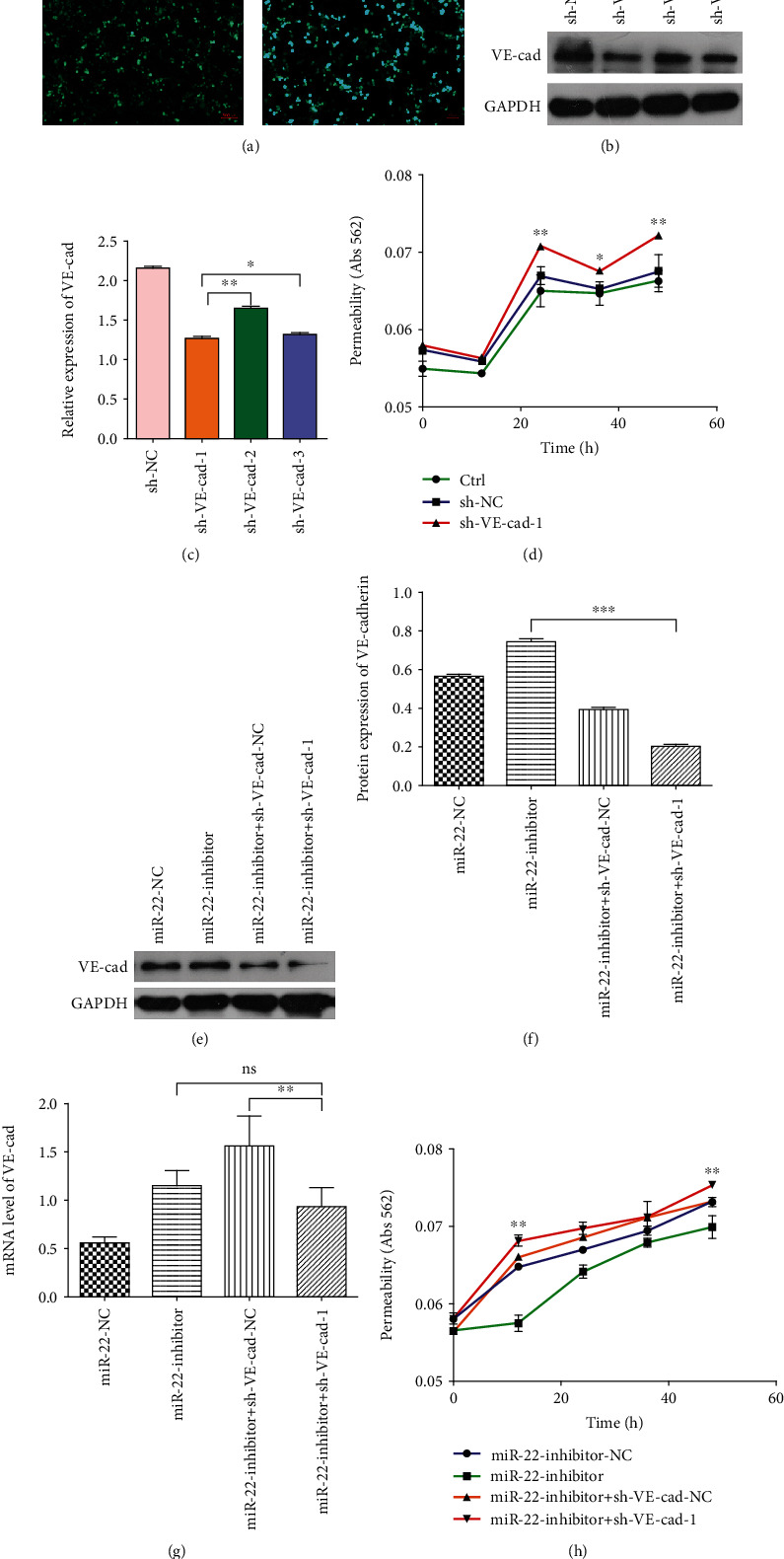
Effects of miR-22-3p on vascular permeability by targeting VE-cadherin. (a) The representative fluorescence figure of HUVECs transfected with VE-cadherin. (b, c) Interference efficiency of VE-cadherin was detected by western blot. One-way ANOVA. ^∗^*p* < 0.05 and ^∗∗^*p* < 0.01. (d) Effects of VE-cadherin on permeability. sh-VE-cad-1 vs. sh-NC. Two-way ANOVA. ^∗^*p* < 0.05 and ^∗∗^*p* < 0.01. (e, f) HUVECs treated as indicated and analyzed by western blot and (g) qRT-PCR. One-way ANOVA. ^∗^*p* < 0.05 and ^∗∗^*p* < 0.01. (h) In vitro permeability assays were performed to measure the effects of miR-22-3p on tubule permeability of HUVECs by targeting VE-cadherin. miR-22-inhibitor+sh-VE-cad-1 vs. miR-22-inhibitor-NC. Two-way ANOVA. ^∗∗^*p* < 0.01. Data are presented as mean ± standarddeviation of at least three independent experiments.

Based on the above findings, the knockdown of VE-cadherin in HUVECs was performed to investigate the effects of VE-cadherin on permeability. Sh-VE-cadherin (1, 2, and 3) and null vector (sh-NC) were transfected to HUVECs (Figure 4(a)). Then, RNA was extracted from the transfected cells, and sh-VE-cad-1 was selected for further analysis (Figures 4(b) and 4(c)). First, sh-VE-cad-1 and sh-NC were transfected to HUVECs, revealing that the permeability of HUVECs enhanced when VE-cadherin decreased (Figure 4(d)). Then, rescue experiments revealed that the protein expression of VE-cadherin promoted by miR-22-3p inhibition could be suppressed by knocking down VE-cadherin (Figures 4(e) and 4(f)), despite no significant changes to VE-cadherin mRNA level was observed (Figure 4(g)), suggesting that miR-22-3p suppressed the translation but not the degradation of VE-cadherin mRNA. Moreover, the tubule permeability suppressed by miR-22-3p inhibition in HUVECs could be restored by sh-VE-cad-1 treatment (Figure 4(h)). Overview, we suggest that miR-22-3p modulates the vascular permeability of HUVECs by directly targeting VE-cadherin.

## 4. Discussion

CRSwNP is known as a common chronic inflammatory disease involving the nasal sinus mucosa. Compared with CRSsNP, the pathological characteristics of CRSwNP include not only the infiltration of inflammatory cells but also interstitial edema [1], which is probably attributed to excessive endothelial permeability [5]. Both this study and our previous researches have demonstrated that NLF-derived exosomes from CRSwNP could increase the permeability of HUVECs, despite the mechanism behind this are still unknown.

Exosomes have attracted much attention for scientific research in recent years. The study on exosomes involves fields such as cancer [7], immunology [25], inflammation [26], and diabetes [27]. In addition, recent researches have revealed that the exosomes derived from NLF can be taken as a new diagnostic indicator of upper respiratory tract disease [28, 29]. As an essential substance required for the communication tool between cells, exosomes perform their functions by transferring some characteristic mRNAs, miRNAs, and proteins. In the meantime, miRNAs play a particularly important role for exosomes. So far, there have been studies suggesting that various exosomal miRNAs, such as miR-25-3p, miR-23a, and miR-103, are involved in the progression of various diseases by affecting vascular permeability [30–32]. However, the changes to exosomal miRNAs content in CRSwNP have yet to be described. In this study, it was found out that miR-22-3p was more significantly expressed in the exosomes derived from CRSwNP than in the control group.

As a member of the miRNA family, miR-22-3p was first identified in tumor cells. In some recent researches, it has been proposed that miR-22-3p can perform some other biological functions. For example, miR-22-3p regulates angiogenesis both in cancer and inflammation [20, 21]. According to Feng et al., the exosomal miR-22-3p derived from mesenchymal stem cells was associated with cardiac ischemic preconditioning [33]. Pofi et al. demonstrated that miR-22-3p played a protective and regulatory role in the renal hemodynamics and function of mice with diabetic kidney disease [34]. Based on these findings, it can be judged that miR-22-3p may be linked to the impact of NLF-derived exosomes from CRSwNP on vascular permeability. To verify the impact of miR-22-3p on permeability may be mediated by exosomes, we quantified the transport of pHNEC-derived miR-22-3p to HUVECs, proving that exogenous miR-22-3p could function in endothelial cells (ECs) via exosomal transport. To determine whether the increase in or reduction to exosomal miR-22-3p is contributory to the changes in permeability, miR-22-3p expression was modulated via miR-22-3p mimic and inhibitor in vitro, which led to the results indicating that the upregulation of miR-22-3p induced an increase in the permeability of HUVECs. Our study pioneered in revealing that exosomal miR-22-3p mediated the changes in permeability and suggesting the potential that exosome-dependent mechanisms mediate the communication of miR-22-3p between respiratory epithelium cells and HUVECs.

The bioinformatic analysis of a region upstream of the miR-22-3p locus indicated multiple putative binding sites for VE-cadherin. Since miRNAs perform their biological functions by activating mRNA degradation and/or inhibiting translation [35], our aim is to further identify VE-cadherin as a direct target of miR-22-3p. It was demonstrated that miR-22-3p could inhibit VE-cadherin expression directly by binding to the specific site in the 3′-UTR of the human VE-cadherin mRNA, which was consistent with the study of Gu et al. [21]. Besides, both western blot and qRT-PCR indicated that the treatment with miR-22-3p-inhibitor or miR-22-3p-inhibitor-exo could enhance the expression of VE-cadherin in HUVECs. Furthermore, rescue experiments revealed that the protein expression of VE-cadherin promoted by miR-22-3p inhibition could be suppressed by knocking down VE-cadherin, despite no significant changes to VE-cadherin mRNA level was observed, suggesting that miR-22-3p suppressed the translation of VE-cadherin mRNA. However, no VE-cadherin mRNA degradation occurred as a result. In summary, these findings suggest that VE-cadherin is a direct target of miR-22-3p.

Importantly, it is widely known that VE-cadherin, an endothelial barrier protein, can regulate the permeability of ECs [36, 37]. In this study, it was found out that VE-cadherin was underexpressed in CRSwNP tissues, and that the reduced expression of VE-cadherin improved the permeability of HUVECs, suggesting the negative regulation of miR-22-3p. This study further confirms that the proosmotic functions of miR-22-3p are attributed to the direct suppression of the secreted antiosmotic factor VE-cadherin within ECs, which may suggest one mechanism of the upregulation of miR-22-3p increasing the endothelial permeability in CRSwNP.

## 5. Conclusions

Overall, this study suggests exosomal miR-22-3p from NLF of CRSwNP enhancing vascular permeability by directly targeting VE-cadherin, which may be one mechanism of the occurrence of CRSwNP.

## Figures and Tables

**Table 1:** Characteristics of study subjects.

	Control	CRSwNP
Total no. of subjects	10 (6 males)	26 (17 males)
Tissue used	IT	NP
Age (y), mean (SD)	42 (12)	44 (16)
Asthma, no.	0	2
Positive Phadiatop result, no.	0	4
Aspirin sensitivity, no.	0	0
Lund-Mackay CT score, mean (SD)	0 (0)	12.8 (3.8)
Lund-Kennedy score, mean (SD)	0 (0)	6.9 (2.2)

## Data Availability

We are willing to share the data of this study. The data used to support the findings of this study are included within the article.
